# Evidence that extra copies of chromosome 1q play a role in the early phases of pancreatic neoplasia

**DOI:** 10.1126/sciadv.adx7501

**Published:** 2026-02-20

**Authors:** Christopher Douville, Jeeun Parksong, Marco Dal Molin, Sarah Graham, Patricia T. Greipp, Ryan Knudson, Samuel Curtis, Yuxuan Wang, Lisa Dobbyn, Maria Popoli, Janine Ptak, Natalie Silliman, Katharine Romans, Christine A. Iacobuzio-Donahue, Alvin P. Makoohon-Moore, Anne Marie Lennon, Michael Goggins, Ralph H. Hruban, Ashley Kiemen, Chetan Bettegowda, Kenneth W. Kinzler, Nickolas Papadopoulos, Laura D. Wood, Bert Vogelstein

**Affiliations:** ^1^ Department of Oncology, the Sidney Kimmel Cancer Center, Johns Hopkins University School of Medicine, 733 N. Broadway, Baltimore, MD 21205, USA.; ^2^ The Ludwig Center, Johns Hopkins University School of Medicine, 733 N. Broadway, Baltimore, MD 21205, USA.; ^3^ The Sol Goldman Pancreatic Cancer Research Center, Johns Hopkins University School of Medicine, 733 N. Broadway, Baltimore, MD 21205, USA.; ^4^ Sidney Kimmel Comprehensive Cancer Center, Johns Hopkins University School of Medicine, Baltimore, MD 21287, USA.; ^5^ Department of Pathology, Johns Hopkins University School of Medicine, 733 N. Broadway, Baltimore, MD 21205, USA.; ^6^ Department of Surgery, Johns Hopkins University School of Medicine, 733 N. Broadway, Baltimore, MD 21205, USA.; ^7^ Department of Laboratory Medicine and Pathology, Mayo Clinic, 200 First Street SW, Rochester, MN 55905, USA.; ^8^ The Howard Hughes Medical Institute, Johns Hopkins University School of Medicine, 733 N. Broadway, Baltimore, MD 21205, USA.; ^9^ Department of Pathology, Memorial Sloan Kettering Cancer Center, 417 Est 68th Street New York, NY 10065, USA.; ^10^ Center for Discovery & Innovation Hackensack Meridian Health, 111 Ideation Way, Nutley, NJ 07110, USA.; ^11^ Department of Medicine, University of Pittsburgh Medical Center, Pittsburgh, PA 15261, USA.; ^12^ Department of Neurosurgery, Johns Hopkins University School of Medicine, 733 N. Broadway, Baltimore, MD 21205, USA.

## Abstract

We searched for oncogenes activated by copy number increases using whole-genome sequencing data of 535 pancreatic ductal adenocarcinomas (PDACs). We found that gains of 1q were the second most common gain, occurring in 213 (39.8%) of PDACs. Single-cell analysis via fluorescence in situ hybridization on 33 cancers confirmed these results. A portion of 1q, rather than the entire 1q arm, was gained in 75 (14.0%) PDACs, allowing us to pinpoint two ~3-megabase regions of 1q that were nearly always gained. These two regions contained *NCSTN* and *PSEN2*, genes that code two subunits of the γ-secretase complex. Evaluation of 267 precancerous lesions revealed that extra copies of *NCSTN* and *PSEN2* were common (49%) in noninvasive neoplasms (high-grade pancreatic intraepithelial neoplasms), which are at relatively high risk for progression to PDACs, but uncommon (6%) in low-grade pancreatic intraepithelial neoplasia lesions, which have low malignant potential. We hypothesize that γ-secretase genes are genetically activated oncogenes in the early phases of pancreatic neoplasia.

## Teaser

Copy number increases of the γ-secretase genes on 1q were identified as candidate oncogenes in pancreatic neoplasms.

## INTRODUCTION

It is estimated that pancreatic ductal adenocarcinomas (PDACs) will strike 67,440 Americans in 2025, and the 5-year survival rate is only 13% (*1*–*3*). These cancers arise in the pancreatic ducts systems and are generally driven by somatic genetic activation of an oncogene (*KRAS*) coupled with genetic inactivation of two or three tumor suppressor genes (*CDKN2A*, *TP53*, and *SMAD4*) (*4*, *5*). Activated oncogenes, including *KRAS*, can be directly targeted by drugs that bind to and inactivate their protein products. This drug-based strategy is generally not applicable to tumor suppressor genes because the proteins encoded by mutant tumor suppressor genes are, in many cases, absent in cancer cells (*6*). Even when such tumor suppressor proteins are present, it is more difficult to pharmacologically activate an inactive protein than to inactivate the function of an active protein (*3*). Thus, virtually all pharmacologic agents for diseases of any type function through inactivation of a protein’s function.

Accordingly, most targeted drugs in clinical use for patients with cancer are designed against oncogenes rather than tumor suppressor genes (*7*). Important examples include Herceptin to treat cancers with activation of the *ERBB2* oncogene, imatinib to treat cancers with activation of the *ABL* oncogene activation, vemurafenib to treat cancers with activation of the *BRAF* oncogene, and, most recently, sotorasib to treat cancers with activation of *KRAS* (*7*). Drugs of this type often generate robust antitumor responses, which typically last for several months. The reason that these drugs do not result in cures of patients with advanced cancers is well understood. Any typical patient with advanced cancer harbors billions of cancer cells, and a small portion (e.g., one in a million) will contain mutations that will likely confer resistance to any targeted agent (*8*). Thus, development of drug resistance is a fait accompli, simply determined by the number of cancer cells and the rate of mutation in cancer cells, which is similar to the rate of mutation in normal cells (*9*).

So how can such targeted agents generate cures in the future? One way is to treat cancers earlier in their evolution when there are fewer neoplastic cells and thus fewer cells harboring resistance-conferring mutations (*10*). This underlines the importance of earlier cancer detection, even in scenarios when the primary tumors can be surgically excised. However, this option is not available for the large number of patients whose cancers are not detected until they are advanced. Mathematical modeling suggests, however, that this scenario is not hopeless. At least in theory, cures of advanced cancers can be achieved when more than one drug is used. The probability that a gene can confer resistance to two drugs is exponentially less than the probability that it can confer resistance to a single drug—as long as the resistance mechanisms do not overlap (*11*). In practice, the idea of treating infectious diseases such as AIDS or tuberculosis with more than one targeted drug is already standard of care and has the identical conceptual rationale (*12*).

This combinatorial drug strategy is challenging to implement for cancer because many cancer types are driven by only one mutant oncogene. For example, PDACs are generally driven by one mutant oncogene (*KRAS*) and two or three mutant tumor suppressor genes (*CDK2NA*, *TP53*, or *SMAD4*) (*5*, *13*). To achieve pharmacologically mediated cures of PDACs, it would therefore be helpful to find a targetable oncogene that is commonly activated in this tumor type. Extensive genetic analysis of pancreatic cancers has thus far not revealed any additional oncogenes that are genetically altered in a large fraction of PDACs other than *c-MYC*, which has been challenging to target (*14*). The failure of unbiased, genome-wide omics approaches to reveal additional oncogenes could be due to the limitations of current sequencing and functional analyses. For example, the activating events could be hidden in the cancer genome’s “dark matter,” such as in the promoters, enhancers, or intergenic regions of protein-encoding genes, in genes that product RNA transcripts but not proteins, or in repeated genomic elements that are difficult to uniquely map (*6*). The activating events could also be epigenetic, involving cell-heritable changes in histone modifications or DNA methylation (*6*, *15*, *16*). Specific epigenetic changes are challenging to causally link to cancer because there are so many nonspecific epigenetic changes in most cancers, with only a minor fraction likely playing a role.

Another type of genetic alteration that is challenging to causally link to cancer is gene duplication. Gene duplications, as well as gene losses, are rampant in cancers—an average of 30% of genes are either lost or gained in a typical solid tumor (*17*–*20*). The great majority of these losses and gains are “passengers” and provide no selective growth advantage to the cancer cell (*21*). However, a panel of isogenic cells that have an extra copy of chromosomes was recently created by CRISPR-based engineering tools (*22*). It was thereby found that extra copies (trisomies) of 1q, 7p, and 8q were required for optimum growth of three different cancer cell lines: one from breast, one from kidney, and one from melanoma. Trisomy of chromosome 1 was particularly notable for its effects on cell growth, and it was proposed that the responsible gene on 1q was *MDM4* in breast cancers and melanomas. Inspired by this study, we attempted to evaluate the presence extra copies of all genes in PDACs in an unbiased manner using whole-genome sequencing (WGS).

## RESULTS

### Copy number alterations in PDACs

Previous studies of PDACs, using karyotypic evaluation of DNA sequencing, have documented heterogeneous chromosomal changes, with the occurrence of trisomies of specific chromosomes differing widely among these studies (*17*–*19*). To evaluate pancreatic tumors in a uniform manner, we purified DNA from the primary tumors of 535 PDACs (derived from 521 patients) (table S1). Primary tumors of the pancreas (as opposed to metastatic lesions in other organs that originated in the pancreas) are often composed of a small number of neoplastic cells embedded within a large number of nonneoplastic cells, such as fibroblasts, inflammatory cells, and endothelial cells. We therefore carefully dissected regions of the primary tumors that had relatively high contents of neoplastic cells and purified DNA from those regions. The DNA was used to make libraries by a technology that allowed high conversion of starting template DNA molecules to library DNA molecules (*23*). Furthermore, each DNA template molecule was barcoded so that it could be uniquely identified (*24*). The libraries were sequenced and had, on average, 125 million [interquartile range (IQR) of 88 million to 146 million] properly paired unique reads. Segmentation and analysis were performed using ichorCNA (Materials and Methods) (*25*).

**Fig. 1.**
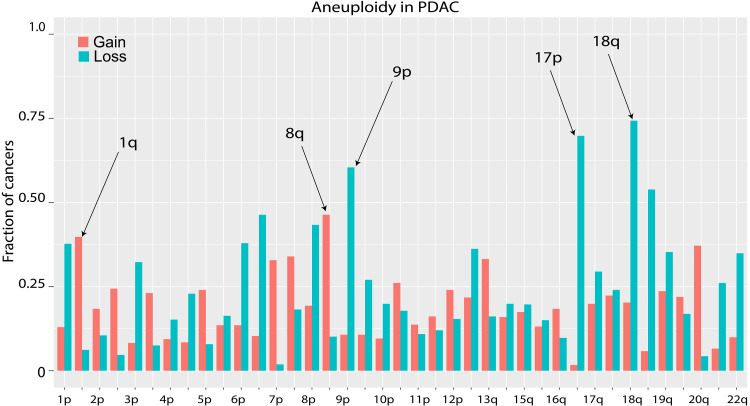
The chromosome arm gains and losses in PDACs were calculated using WGS. The *y* axis denotes the fraction of tumors with a gain (or loss) for the indicated chromosome arm on the *x* axis.

A summary of chromosome gains and losses in these 535 PDACs is shown in Fig. 1 and table S1. Higher-resolution copy number calls based on segmentation of 500-kb intervals is shown in table S2A. Raw ichorCNA outputs and genome-wide plots for all samples are available at https://zenodo.org/records/17080198 (DOI: 10.5281/zenodo.17080197). The chromosome arms with the highest fraction of losses were chromosomes 9p (*CDK2NA*), 17p (*TP53*), and 18q (*SMAD4*), which were lost in 60.4, 69.9, and 74.2% of the PDACs, respectively. The predominance of these chromosome arms was expected given that the predominant known PDAC driver genes are *CDKN2A*, *TP53*, and *SMAD4*, located on chromosomes 9p, 17p, and 18q, respectively. We then compared our results to The Cancer Genome Atlas (TCGA), which evaluated aneuploidy in 184 pancreatic adenocarcinoma the Affymetrix SNP 6.0 and GISTIC2 algorithm (*26*). TCGA reported slightly lower rates of loss on these chromosomes: 42% (9p), 46% (17p), and 62% (18q).

With respect to gains, two chromosome arms stood out: Chromosomes 1q and 8q [hereinafter denoted (1q and 8q)] were gained in 39.8 and 46.7% of the 535 PDACs, respectively. TCGA estimates were again slightly lower than ours (1q, 28%; 8q, 25%) (*26*). In those cancers with gains of 1q, the copy number of 1q varied from 3 to 7 among different cancers. Analogously, in those cancers with gains of 8q, the copy number of 8q also varied from 3 to 7 among different cancers. We asked whether 1q and 8q gains as well as other common chromosomal gains were mutually exclusive or independent events. Although some deviations from random partitioning were apparent (table S1), the effects sizes were small, suggesting minimal if any biological selection.

We next asked whether the choice of bioinformatic algorithm would change the interpretation of aneuploidy in PDACs. We repeated the analysis using QDNAseq to identify copy number alterations (Materials and Methods) (*27*, *28*). A detailed list of the predicted copy number calls based on segmentation for all 500-kb intervals throughout the genome are included in table S2B. Raw QDNAseq outputs and genome-wide plots for all samples are available at https://zenodo.org/records/17080198 (DOI: 10.5281/zenodo.17080197). The two most common gains were again 1q (42.2% in QDNAseq versus 39.8% inichorCNA) and 8q (45.8% versus 46.7%), whereas the most common losses were 9p (69.9% versus 69.9%), 17p (73.6% versus 69.8%), and 18q (81.7% versus 74.2%). The two algorithms therefore produced very similar results, with one notable exception: A subset of the tumors that did not have detectable levels of aneuploidy with ichorCNA had low but detectable levels of aneuploidy when assessed with QDNAseq (table S2, A and B).

### Fluorescence in situ hybridization

The WGS data show that 1q gains are common among PDACs but do not provide data about the distribution of gains among the cells of a tumor, i.e., how many neoplastic cells within the cancer contain gains. The 1q gains reported in Fig. 1 and table S1 additionally reflect relative gains, i.e., relative to all other chromosomal arms in the same sample, rather than absolute gains. For example, if the cells are uniformly tetraploid through genome doubling, this would be impossible to discern with the technique we used—all chromosome arms would appear to have a copy number of 2. Although it is possible to infer absolute gains from WGS data, it is bioinformatically challenging (*29*, *30*).

To provide data about cellular distribution and the absolute number of 1q chromosomes per pancreatic cancer cell, we used fluorescence in situ hybridization (FISH) with a labeled probe from a gene located in the middle of chromosome 1 (*ABL2*, located at *1q25*). In each case, 50 to 100 nuclei within formalin-fixed and paraffin-embedded (FFPE) sections were evaluated by two individuals in a blinded manner (Materials and Methods). As controls for these experiments, we evaluated acinar regions of seven patients with PDAC using FFPE sections of the surgically excised normal pancreas distant from the cancer itself. This ensured the absence of cancer cell nuclei in the entire evaluated sample. We additionally evaluated normal acinar regions, or the ducts themselves, of two other patients with PDAC. In these two patients, pancreatic cancer cells were observed in the FFPE sections but separated by at least 2 mm from the evaluated nonneoplastic acini and ducts. In the nonneoplastic cells on these sections, the average number of 1q fluorescent signals per nonneoplastic nucleus ranged from 1.8 to 2.1, and only 3.4% of 900 nuclei contained more than three or more fluorescent signals (table S3).

**Fig. 2.**
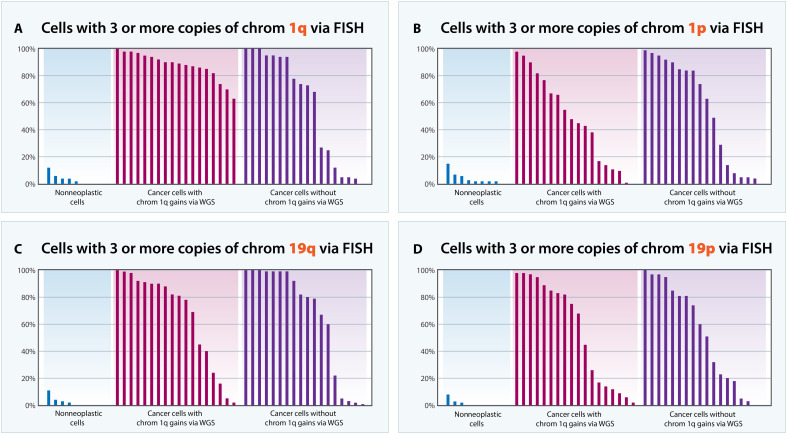
PDACs were evaluated using FISH. Sections of primary PDACs were evaluated with clinically certified hybridization probes. In each panel, the *y* axis denotes the percentage of cells with three or more copies of chromosomes 1q (**A**), 1p (**B**), 19q (**C**), and 19p (**D**). As assessed by WGS, there were 18 tumors in which chromosome 1q was gained [purple bars in the middle of (A) to (D)] and another 18 cases in which chromosome 1q was not gained [purple bars at the right of (A) to (D)]. In all tumors, adjacent sections were used to identify cancer cells (i.e., neoplastic cells) using standard histopathologic criteria, and the cancer cells in these sections were scored by FISH. In a subset of cases, the nonneoplastic cells in the sections of tumors (e.g., normal ductal epithelium or stromal cells) were identically scored by FISH as controls [blue bars at the left of (A) to (D)] as controls.

**Fig. 3.**
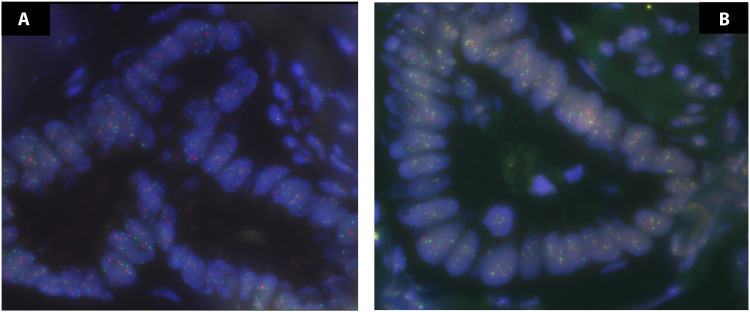
Representative images of the FISH analysis for CBPANC18 (PDACs). (**A**) FFPE section hybridized with probes for *1p36.3* (red signal) and *1q25* (green signal). (**B**) adjacent section hybridized with probes for *19p13* (green signal) and *19q13* (red signal). The nuclei are stained with 4′,6-diamidino-2-phenylindole (DAPI) (blue). The small nuclei at the top right corner of both pictures are nonneoplastic cells (diploid), whereas most other cells are PDAC ductal structures (aneuploid).

We next evaluated neoplastic cell nuclei in FFPE sections of cancers from 33 patients with PDAC, 15 with 1q gains evident in WGS and 18 without 1q gains (table S1). FISH confirmed that all 15 patients with WGS-defined chromosome gains in their cancers had extra copies of 1q. The average number of 1q fluorescent signals ranged from 2.9 to 4.9 in these 15 patients, and most nuclei in each patient contained at least three fluorescent signals (table S3 and summarized in table S4; summary graphed in Fig. 2 and FISH examples in Fig. 3). We found that some of the patients with pancreatic cancer in whom WGS did not reveal copy number alterations also had extra copies of 1q. In 11 of these 18 patients, most nuclei contained at least three 1q fluorescent signals per nucleus (Figs. 2 and 3 and tables S3 and S4). A subset (*n* = 4) of the discordant tumors could be attributed to low tumor cellularity and the technical limitations reliably identifying copy number changes at low admixtures. Most discordances (*n* = 6), however, stem from the limitation of WGS algorithms identifying gains or losses relative to the basal copy number of all changes rather than absolute copy number.

Our FISH observations are consistent with conclusions from previous studies of cancers, including pancreatic cancers, showing that whole-genome duplication is a common event (*29*–*31*). To provide further evidence for whole-genome duplication in the cancers in our cohort, we evaluated three other probes with FISH, from chromosomes 1p, 19p, or 19q. These probes were chosen because they have been extensively used and clinically validated in brain tumors for the detection of the 1p/19q translocations typical of oligodendrogliomas (*32*, *33*). We found that 72, 68, and 72% of the 37 PDAC samples had at least three copies of chromosomes 1p, 19p, or 19q in at least 20% of their neoplastic nuclei. Similarly, 62, 62, and 70% of the 37 PDAC tumors contained at least four copies of chromosomes 1p, 19p, or 19q in at least 10% of their neoplastic nuclei. Gains of 1p, 1q, 19p, or 19q of this magnitude (i.e., most cells) were not found in any of the 11 nonneoplastic control samples (Fig. 2 and table S5).

### Copy number alterations in the precursors to PDACs

The great majority (~90%) of PDACs are believed to develop from pancreatic intraepithelial neoplasia (PanIN) lesions (*34*, *35*). These lesions are located in the ducts and can be classified into low-grade (LG) and high-grade (HG) based on the degree of histologic dysplasia, e.g., their resemblance to normal pancreatic ductal cells, including the size, shape, and position of the cells and the nuclei within the cells. PanINs cannot be detected by imaging because they are <0.5 cm in diameter. Instead, they can only be observed through microscopic evaluation of excised pancreata. There is an average of ~1000 LG PanINs in individuals older than 65 years, and their danger of progressing to malignancy is close to nil (*36*). In contrast, HG PanINs are much less common and are thought to have a relatively high chance of progressing to a PDAC. More than 90% of all PanINs contain mutations in *KRAS* genes, and the genetic and epigenetic alterations responsible for progression from LG PanINs to HG PanINs are an active area of investigation (*5*, *37*).

**Fig. 4.**
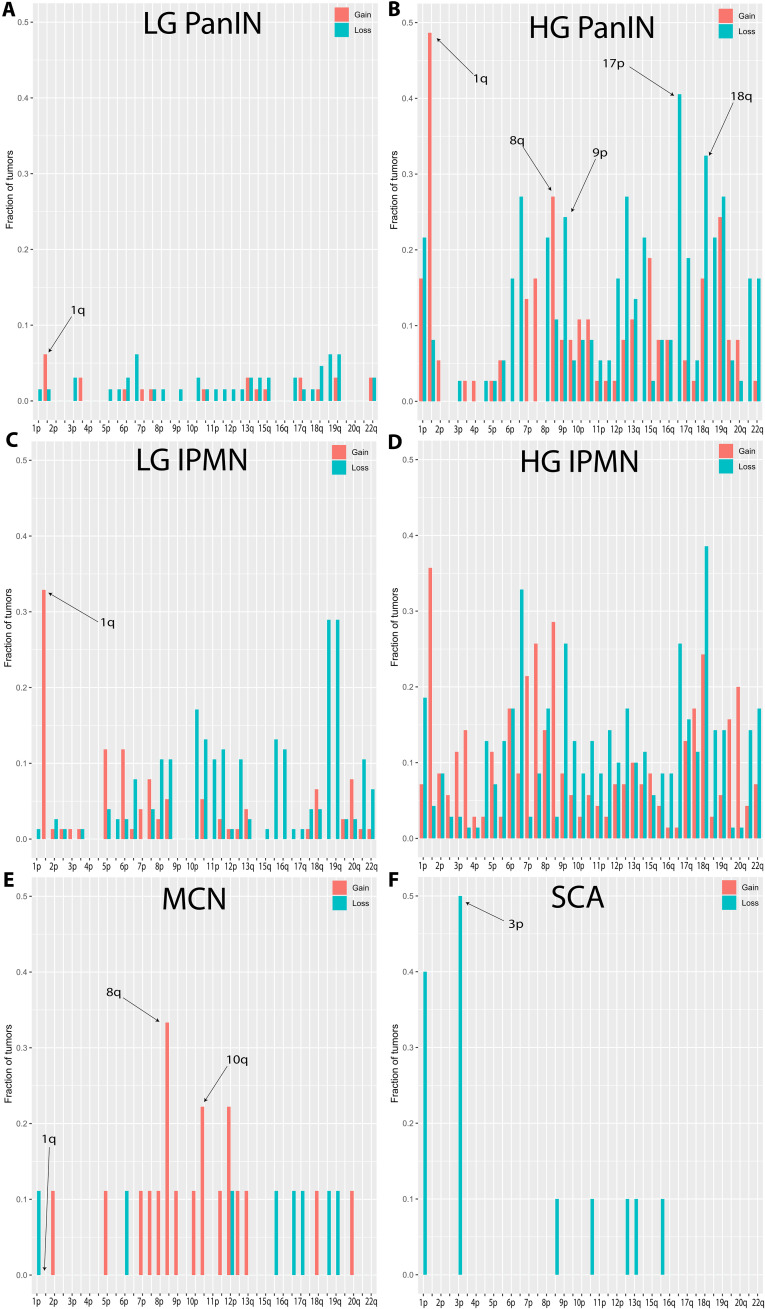
The chromosome arm gains and losses in various types of pancreas neoplasia were calculated using WGS. In each panel, the *y* axis denotes the fraction of tumors with a gain (or loss) for the indicated chromosome arm on the *x* axis.

Because PanINs are so small, microdissection of the lesions was required to obtain a high neoplastic fraction in the tissues used for purifying DNA. As controls, we dissected nonneoplastic regions from 45 sections of pancreas containing LG or HG preneoplastic lesions. None of these 45 contained any chromosome gains or losses (table S1). As expected, in 65 LG PanINs derived from 49 patients, aneuploidy was also infrequent: 81% had no chromosome gains or losses (*38*). The most frequently altered chromosome arm in LG PanINs was 1q: 4 (6%) of the 65 had gained 1q (Fig. 4A and table S1). In these four, one had no other gains or losses of any chromosome arm, one had a loss of 6q, another had a gain of chromosome 7 (both p and q arms), and the other (PIN 108 S2) was highly aneuploid, with a total of 26 gains or losses, including losses of 9p, 17p, and 18q (table S1). This LG PanIN was adjacent to a HG PanIN, with which it shared the gain of 1q and the losses of 9p, 17p, and 18q, suggesting an evolutionary relationship (table S1).

The evaluation of HG PanINs revealed a picture different from either LG PanINs or PDACs (Fig. 4B). The most prevalent altered chromosome arm was again 1q, gained in 49% of the 37 lesions (derived from 28 patients) studied (Fig. 4B). In the 18 HG PanINs with gain of 1q, two had no gains or losses of any other chromosome arm, and seven (39%) did not gain 8q or lose 9p, 17p, or 18q. In comparison, only 3 (1.4%) of 212 PDACs with chromosome 1q gain did not gain 8q or lose 9p, 17p, or 18q (table S1).

Most of the PDACs that do not originate in PanINs are believed to originate from intraductal pancreatic mucinous neoplasms (IPMNs) (*5*, *39*). We were able to evaluate 76 LG IPMNs (derived from 44 patients) via WGS. In these lesions, the most frequent chromosome arm change by far was 1q gain, occurring in 25 (33%) of 76 tumors (Fig. 4C)—considerably higher than in LG PanINs (7%; Fig. 4A). In 12 (48%) of the LG IPMNs with 1q gain, no gains or losses of any other chromosome arm were identified (table S1). All 25 (100%) of the LG IPMNs with 1q gain did not lose 9p, 17p, or 18q, and only 2 (8%) gained 8q (table S1).

We also evaluated 70 HG IPMNs (derived from 21 patients) and found that 1q gains were about as common in these lesions (36%; Fig. 4D) as they were in LG IPMNs (33%; Fig. 4C). However, other chromosome gains and losses were much more frequently observed in HG IPMNs than in LG IPMNs. Of the 25 HG IPMNs with 1q gain, only one (4%) did not have gains or losses of at least one other chromosome (table S1). In the 25 HG PanINs, seven (28%) did not gain 8q or lose 9p, 17p, or 18q—far higher than the 1.4% of PDACs which did not gain or lose these chromosome arms (table S1).

PDACs can also, although uncommonly, originate from mucinous cystic pancreatic neoplasms (MCNs) (*39*). These are distinguished from PanINs and IPMNs by epidemiology, histopathology, and the genes most commonly mutated. For example, MCPNs usually occur in middle-aged females, contain ovarian-type stroma by histopathologic definition, and often contain mutations in *RNF43*. We were able to evaluate nine dissected MCN lesions (derived from nine patients). None had gains of 1q, although four (44%) of the nine had gains or losses of other chromosome arms (Fig. 4E and table S1).

Last, we evaluated carefully dissected regions from 10 serous cystadenomas (SCAs) (derived from 10 patients), benign tumors that do not progress to malignancy (*39*). None had gains of 1q, although 5 (50%) of the 10 had gains or gains or losses of other chromosome arms (Fig. 4F and table S1). All five of the SCAs with gains or losses of other chromosomes had lost 3p—the site of the *VHL* gene known to be a driver gene in this tumor type (*40*, *41*).

The WGS results of the eight types of pancreatic samples we studied are graphed in. The average number of chromosome arms gained or lost in these lesions varied from zero to 17. No 1q gains were found in normal tissues, MCPNs, or SCAs. Chromosome 1q gains were found in 7, 49, 36, 33, and 40% of LG PanINs, HG PanINs, LG IPMNs, HG IPMNs, and PDACs, respectively. In those PDACs that did harbor a 1q gain, nearly all cases also had gains of 8q or losses of chromosomes 9p, 17p, or 18q (99%). However, in HG precursor lesions with 1q gain, additional gains or losses of these four chromosome arms were observed in fewer cases (67%, *P* < 0.0001 by two-tailed Fisher’s exact test; table S1). In LG precursor lesions with 1q gain, additional gains or losses of these other chromosome arms were even less frequent (10%, *P* < 0.0001 by two-tailed Fisher’s exact test; table S1).

### Localization of the regions on 1q containing candidate driver genes

In heritable diseases, linkage analysis can identify candidate genes that may be responsible for the predisposition to the disease. In cancer, which is mainly driven by somatically acquired mutations, the analog of linkage is the identification of gene(s) that are most frequently associated with the genetic alteration of interest in sporadic tumors (*42*). For example, this strategy led to the identification of *TP53* as the tumor suppressor gene inactivated in most human cancers (*43*). We attempted to apply this strategy to identify candidate driver gene(s) on 1q.

**Fig. 5.**
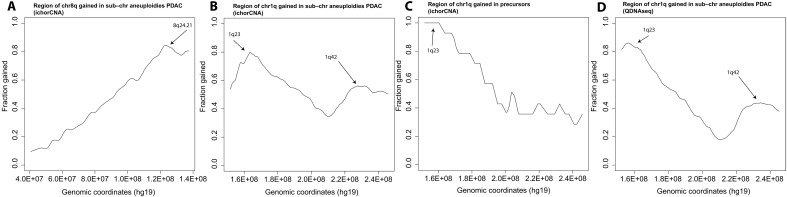
Regions gained in tumors with subchromosomal gains. (**A**) Chromosome 8q in PDACs (ichorCNA). (**B**) Chromosome 1q in PDACs (ichorCNA). (**C**) Chromosome 1q in PDAC precursors (PanINs or IPMNs) (ichorCNA). (**D**) Chromosome 1q in PDACs (QDNAseq). In each panel, the *y* axis denotes the fraction of tumors with gains at the indicated chromosomal position on the *x* axis.

We began with a well-known target of chromosome gain, i.e., 8q. It is believed that *c-MYC* gene is the target of 8q gains in multiple cancer types. 8q is the most frequently gained chromosome arm in PDACs (Fig. 1), although it is not as often gained in PDAC precursors (Fig. 4). Our WGS data showed that 50% (*n* = 124) of the PDACs that gained 8q gained the entire 8q arm, whereas the remaining 50% (*n* = 123) gained only a subset of 8q genes. Cancers that gained the entire arm obviously cannot be used to identify the presumptive driver gene on that chromosome arm. However, evaluation of the 123 PDACs that gained only a subset of the genes on the arm can address this question. On 8q, there was a single broad peak centered at *8q24.21*, spanning positions 127,300,001 to 131,500,000 (Fig. 5A). This peak includes *c-MYC* (128,747,681 to 128,755,197). Moreover, 93% of the cancers that gained all or any part of 8q gained *c-MYC*.

Analogously proceeding to 1q, the WGS data showed that 139 (66%) of the PDACs that gained 1q gained the entire 1q arm, whereas the remaining 73 (34%) gained only a subset of 1q genes. Unlike the single major peak on 8q, there were two major peaks on 1q: one centered at *1q23.2* (159,100,001 to 160,500,000) and the other centered at *1q42.13* (227,000,001 to 230,700,000) (Fig. 5B). Ninety-one percent (*n* = 192) of the cancers that gained any part of 1q (including the entire 1q) gained the *1q23.2* locus and 85% (*n* = 180) of the cancers that gained any part of 1q gained the *1q42.13*. Considering only the 212 PDACs that had gained extra copies of any part of 1q, 77% (*n* = 164) gained both the *1q23.2* and *1q42.13* loci, 13% (*n* = 28) gained the *1q23.2* locus but not the *1q42.13* locus, 8% (*n* = 16) gained the *1q42.13* locus but not the *1q23.2* locus, and 2% (*n* = 4) gained neither. The WGS data on PDAC precursor lesions highlighted the same two regions of 1q, but the number of tumors was much smaller and the localization therefore of lower resolution (Fig. 5C).

### Identification of candidate oncogenes on 1q

The critical region on *1q23.2* contained 38 transcribed genes, 32 of which encode proteins (fig. S1A) (*44*, *45*). To determine the most likely genes responsible for the putative selective growth advantage conferred by this region of 1q, we evaluated the correlation between gene gain and gene expression in TCGA database (*46*). This analysis only examines the impact of copy number alterations without examining the impact of other genetic or epigenetic alterations on transcription levels. There were nine genes in which the level of expression and copy number were correlated in a statistically significant manner (*P* < 1 × 10^−5^; red font in fig. S1A; details in table S5). Notable among these was *NCSTN*, encoding a component of the γ-secretase complex.

The second critical region on *1q42.13* (chr1: 227,000,001 to 230,700,000; fig. S1B) contained 61 transcribed genes, 33 of which encode proteins. To determine the most likely genes responsible for the putative selective growth advantage conferred by this region of 1q, we similarly evaluated the correlation between gene gain and gene expression in TCGA database (*46*). There were 14 genes in which the level of expression and copy number were correlated in a highly statistically significant manner (*P* < 1 × 10^−5^; red font in fig. S1B; details in table S6). Notable among these was *PSEN2*, encoding another component of the γ-secretase complex.

### Relationship of 1q gain to common genetic alterations in PDAC precursor lesions

The average depth of the WGS was 121 million unique properly paired reads per sample, corresponding to ~5× coverage, and this was insufficient to reliably evaluate subtle mutations, such as single base substitutions, insertion, or deletions, in the genes driving PDAC development. We therefore designed primers to evaluate 37 amplicons (table S7), considering both the Watson and Crick strands, to detect subtle mutations in commonly altered regions of *KRAS*, *NRAS*, *GNAS*, *CDK2NA*, *SMAD4*, and *TP53* in a highly specific manner (Materials and Methods). This analysis could be performed on 138 DNA samples from carefully dissected precursor lesions, including 31 LG PanINs, 24 HG PanINs, 43 LG IPMNs, and 40 HG IPMNs. Mutations in either *KRAS* or *GNAS* were identified in all 138 samples (100%; table S8), as expected from prior studies, suggesting that mutations in these two genes initiate pancreatic ductal neoplasia. Because all lesions had mutations in these two genes, there could obviously be no correlation between 1q gain and mutations in *KRAS* or *GNAS*. However, we could evaluate how often subtle mutations in *CDKN2*, *TP53*, or *SMAD4* occurred. We found that most (76%) of the 38 precursor lesions with 1q gain did not have detected mutations in *CDKN2*, *TP53*, or *SMAD4*, consistent with the idea that 1q gains can precede these other genetic alterations during tumor progression (table S9). Conversely, most (65%) of the 26 precursor lesions in which *CDKN2*, *TP53*, or *SMAD4* mutations were detected did not harbor 1q gains, consistent with the idea that imbalanced 1q gains are not required for tumor progression (table S9).

## DISCUSSION

Our results provide evidence that

1) 1q gains are the most common chromosomal observation in the early stages of pancreatic neoplasia.

2) 1q gains often occur before alterations in previously known cancer PDAC driver genes at either the chromosomal or intragenic point mutation levels.

3) Two regions on 1q can be pinpointed as harboring 1q driver genes.

4) Both of these two regions contain genes encoding γ-secretase subunits.

5) γ-Secretase expression is correlated with these 1q gains.

There are many previous studies of aneuploidy in PDACs using either microarrays or WGS [reviewed in (*47*)]. For example, Mullen *et al.* studied multiple independent regions of 96 PDACs and found that higher number of mutations in the founder cells giving rise to PDACs was correlated with poor survival (*31*). Waddell *et al.* evaluated 100 PDACs and highlighted that structural rearrangements could be used to classify the tumors into four subtypes that were associated with therapeutic responsiveness (*48*). Hirada *et al.* evaluated 27 PDACs and highlighted amplification of the *SKAP2/SCAP2* gene on chromosome *7p15.2*, proposing it as a candidate oncogene (*49*). Hata *et al.* evaluated 7 PDACs, 11 HG PanIN, and 2 LG PanIN lesions, with results that were not inconsistent with those reported in our study (*50*). Notta *et al.* studied 107 PDACs and found that 45% displayed changes in copy number consistent with whole-genome duplication and highlighted that *TP53* mutation was correlated with this duplication (*30*). The importance of *TP53* mutation with respect to whole-genome duplication was further emphasized by the study of Baslan *et al.*, in which extensive evaluations of mouse models of PDACs revealed that p53 inactivation preceded other chromosomal changes (*51*). The results of our study did not add support to the idea that p53 mutation precedes chromosome 1q duplication. Most (76%) of the 38 precursor lesions with 1q gain in our study did not have mutations in *TP53* detectable by targeted deep sequencing (tables S1, S8, and S9). Of the nine lesions that did have *TP53* mutations, six had loss of chromosome 17p, as expected given the association of *TP53* mutations with chromosome 17p loss (*43*). It will be important in future studies to determine whether this discrepancy is a reflection of the difference between human cancers and the mouse models or whether 1q gain (which was not a focus of the Baslan *et al.* study) is an exception to the hypothesis that p53 inactivation precedes chromosome gains in general. It will also be informative to see whether chromosome 1q gain or γ-secretase genes play any role in mouse PDAC development. If it did, a wealth of experimental possibilities would be opened given the power of genetically engineered mouse models to reveal insights into the tumorigenic process that cannot be discerned from evaluation of human tissue biopsies.

None of the studies noted in the above paragraph had highlighted 1q as the location of a major oncogene. We believe that our study was able to single out 1q and γ-secretase genes for several reasons. First, we analyzed a large number of precursor lesions in addition to cancers; the 1q gains stood out much more in precursor lesions than in cancers because of the much more complex karyotypes of cancers. Second, the entire 1q arm is gained in most pancreatic lesions with any part of 1q gained. To sublocalize the region of chromosome 1q that is presumably driving the 1q gain requires evaluation of a large number of tumors, only a subset of which will have only a portion of 1q gained. We were able to evaluate a total of 802 neoplastic lesions of the pancreas, including 535 cancers and 267 precursors, substantially greater than reported in previous studies.

Our results also pose a variety of intriguing and as yet unanswered questions, which can be considered weaknesses of our study.

### Functional studies

We do not provide any functional support for the hypothesis that γ-secretase activation by gene duplication is an oncogenic event. Our evidence is all based on genetic associations. However, it has been shown that reversion of 1q gains in breast cancers, kidney cancers, and melanomas have major effects on tumor cell growth (*16*, *47*)—these observations are what inspired our study. Analogous functional studies on pancreatic cancers should be performed in the future to support our conclusions. However, the most compelling evidence that genes altered by point mutations are actually drivers is genetic rather than functional (*6*). Such genetic evidence is based on data showing that point mutations in a candidate gene occur much more commonly than those in all other genes in the genome so are statistically well controlled. By analogy, 1q gains are observed much more frequently in PDAC precursor than any other chromosome gain or loss, often in the absence of any other chromosome changes—providing evidence that 1q gains drive the neoplastic process and are not simply passengers.

### The culprit genes

If one accepts that 1q gain is a driving genetic alteration, what is the gene(s) responsible for its oncogenic effects? In other cancer types, 1q genes, such as *MDM4* (*52*), *MCL1* (*53*), *AKT3* (*54*), and *KDM5B* (*55*), as well as γ-secretase (*42*), have been proposed. Our study is able to localize particular regions of 1q to more definitively identify the candidates. This localization was only feasible because of the large number of samples we evaluated, given that subchromosomal 1q gains are uncommon in any cancer type. We found two regions of 1q to undergo particularly frequent gains: one at *1q23.2* and one at *1q42.13*. Evaluation of transcription patterns supported our hypothesis that γ-secretase components encoded by *NCSTN* and *PSEN2* are the culprits in these regions. Moreover, 98% of PDACs having extra copies of any part of 1q had extra copies of either *NCSTN* or *PSEN2* (tables S1 and S3). However, there are other genes that are differentially expressed in PDACs harboring 1q gains compared to those without 1q gains (fig. S1). Future functional studies can provide support for the hypothesis that any of these genes, alone or in combination, are responsible for the selective growth advantage afforded by 1q gain. Our nomination of γ-secretase genes as the culprits for the PDAC drivers on 1q is supported by recent studies on liver and breast cancers, which provided functional evidence for the role of γ-secretase genes in that tumor type (*42*, *56*).

### Substrates for γ-secretase

Assuming that γ-secretase genes are the culprit 1q-driving events, what is the substrate for the γ-secretase protease complex? Strong evidence that this substrate is Notch has been provided through pathway analysis (*57*, *58*). Watson *et al.* also noted that subtle mutations of Notch itself are found in breast cancers, strengthening the evidence in support of Notch (*42*). However, Notch is not mutated in PDACs. Also, γ-secretase is known to cleave many other proteins. Among these, one of the most intriguing is E-cadherin. Prior studies have shown that HG PanIN cells have the capacity to migrate through the ducts and initiate new lesions (*59*), whereas LG PanIn cells do not (*36*). The disruption of cell-cell contact is critical for such migration (as well as for metastasis), and E-cadherin is one of the most important proteins mediating intercellular attachments and regulation of cell growth (*60*). E-cadherin a known tumor suppressor gene (*61*) and well-documented substrate of γ-secretase, In breast cancers, gain of 1q is often accompanied by loss 16q, wherein E-cadherin resides. We therefore speculate that a critical substrate of γ-secretase in both breast and PDACs is E-cadherin.

### The frequency of γ-secretase gene duplication

The WGS data show that duplication of γ-secretase genes occur in ~40% of cancers and HG PanINs. However, the FISH data show that these genes are duplicated, likely through whole-genome duplication, in a much higher fraction of PDACs (table S4). As noted in the results, the reason for this discrepancy is likely to that whole-genome duplication cannot be easily detected in WGS of relatively pure cell populations, whereas FISH has this capacity. These observations bring up several important questions. First, can an increase in 1q in a tetraploid context result in a selective growth advantage to the cell? It is possible that, when all nuclear genes are duplicated, no imbalance of gene expression occurs and no effect on cell growth occurs. This possibility has not been evaluated in a previous study in which the effects of 1q gain were evaluated (*22*). Second, does tetraploidy occur before, or only after, the imbalanced duplication of 1q that is detectable by WGS? This question about the timing of whole-genome doubling versus imbalanced 1q gains in the various stages of pancreatic neoplasia can be addressed by in situ studies of precursor lesions of various grades, combined with WGS and karyotyping, in the future. It has recently been shown that ~3% of morphologically normal breast epithelial cells contain duplications of 1q genes (*62*, *63*).

### Therapeutic implications

Γ-Secretase genes came to the forefront of research because of their involvement in Alzheimer’s disease, not because of their involvement in cancer. It was thought that inhibiting the γ-secretase enzyme would inhibit cleavage of the amyloid precursor protein (APP) and reduce amyloid plaque formation (*64*–*66*). Numerous inhibitors or modulators of γ-secretase activity have therefore been developed for the treatment of Alzheimer’s disease. Some of these drugs have been tested in patients with cancer, with relatively little activity observed in most studies (*66*). One exception is the recent approval of nirogacestat for the treatment of aggressive desmoid tumors (*67*). Notably, chromosome 1q gains are the most prominent karyotypic feature of desmoid tumors (*68*) and it is conceivable that those desmoid tumors, which respond to nirogacestat, are precisely those than harbor 1q gains. Perhaps nirogacestat or related drugs can be tested for the treatment of PDACs that contain extra copies of γ-secretase genes and particularly high expression of the γ-secretase protease complex. Combining γ-secretase inhibitors with KRAS inhibitors is a particularly attractive strategy because genetic alterations of both KRAS and γ-secretase genes are so commonly found in this type of cancer. Last, identification of the most important substrates of γ-secretase in PDACs could lead to drugs that preferentially inhibit the action of those substrates, rather than all substrates of γ-secretase, with less systemic toxicity.

## MATERIALS AND METHODS

### Patient samples

This study was approved by the Institutional Review Boards for Human Research at Johns Hopkins Hospital and other participating institutions (protocols NA_00001584, NA_00017879, and IRB00044588) in compliance with the principles of the Declaration of Helsinki and the Health Insurance Portability and Accountability Act. All patients provided written informed consent. All samples were deidentified immediately following collection. A subset of the samples described in table S1 have been included in prior publications for other purposes, but none of the sequencing or FISH data reported in this study have been previously published. Sections of frozen or FFPE tumor tissues were either macro- or microdissected and DNA purified, as described in (*59*, *69*–*71*). All samples were reviewed and graded by an expert pancreatic pathologist based on histological features.

### Library construction

We developed a library preparation workflow that can efficiently recover input DNA and simultaneously incorporate double-stranded molecular barcodes (*23*). In brief, libraries were prepared using an Accel-NGS 2S DNA Library Kit (Swift Biosciences, 21024) with the following critical modifications: (i) DNA was pretreated with 3 U of USER enzyme (New England Biolabs, M5505L) for 15 min at 37°C to excise uracil bases; (ii) the SPRI bead/PEG NaCl ratios used after each reaction were 2.0×, 1.8×, 1.2×, and 1.05× for end repair 1, end repair 2, ligation 1, and ligation 2, respectively; (iii) a custom 50 μM 3′ adapter was substituted for reagent Y2; and (iv) a custom 42 μM 5′ adapter was substituted for reagent B2. Libraries were subsequently polymerase chain reaction (PCR) amplified in 50-μl reactions using primers targeting the ligated adapters. The following reaction conditions were used: 1× NEBNext Ultra II Q5 Master Mix (New England Biolabs, M0544L), 2 μM universal forward primer, and 2 μM universal reverse primer. Libraries were amplified with 8 or 11 cycles of PCR, depending on how many experiments were planned, according to the following protocol: 98°C for 30 s, cycles of 98°C for 10 s, 65°C for 75 s, and hold at 4°C. If eight cycles were used, the libraries were amplified in single 100-μl reactions. If 11 cycles were used, the libraries were divided into eight aliquots and amplified in eight 50-μl reactions, each supplemented with an additional 0.5 U of Q5 Hot Start High-Fidelity DNA Polymerase (New England Biolabs, M0493L), 1 μl of 10 mM deoxynucleoside triphosphates (Deoxynucleoside triphosphates; New England Biolabs, N0447L) and 0.4 μl of 25 mM MgCl_2_ solution (New England Biolabs, B9021S). The products were purified with 1.8× SPRI beads (Beckman Coulter, B23317) and eluted in EB buffer (Qiagen).

### Aneuploidy analysis

Library DNA was amplified in 50-μl reactions in Ultra Q5 with primers at 2 μM for seven cycles with the following conditions: 98°C for 30 s and then seven cycles of 98°C for 10 s to denature, and 65°C for 75 s to anneal and extend. WGS libraries were sequenced on a NovaSeq 6000 with paired-end 2 × 100–base pair (bp) reads. Cutadapt was used to trim 27 bp from both reads (*72*), and BWA-MEM was used to align reads to the hg19 genome. Duplicate molecules were marked and removed using samtools (*73*). Reads with a bowtie2 alignment quality score of >10 were binned into 500-kb intervals and counted. IchorCNA (*25*) was then used to perform GC correction and segmentation using the following parameters: “—chrs “c (1,2,3,4,5,6,7,8,9,10,11,12,13,14,15,16, 17,18,19,20,21,22) –normalPanel.” Two normal panels were generated—one for fresh frozen samples (*n* = 9) and one for FFPE samples (*n* = 5). The copy number calls (G, gain; N, neutral; L, loss) during ichorCNA segmentation were reported in table S2A. As recommended in the ichorCNA best practices on GitHub, only samples with a GC correction Mean Absolute Deviation of <0.15 were used in this study. QDNAseq was also used to perform GC correction, segmentation, and calling of copy number alterations throughout the genome (*27*, *28*). We include the QDNAseq expected and observed variance as well as the number of calculated segments in table S1. Samples that have much larger observed variance than the expected variance may suggest a sample with poor quality. The copy number calls (G, gain; N, neutral; L, loss) during QDNAseq segmentation were reported in table S2B. A full set of files (log ratios, segmentation files, plots, and .RDS) for both ichorCNA and QDNAseq analyses are publicly available at https://zenodo.org/records/17080198 (DOI: 10.5281/zenodo.17080197). In our analysis, we defined a full arm gain by counting the number of 500-kb intervals on a particular chromosome arm with a predicted copy number of >2. If the number of gained intervals was greater than or equal the number of 500-kb intervals on the chromosome arm—3 intervals, it was considered a full arm gain (e.g., no more than 3 nongained intervals were permitted on the arm to be considered a full arm gain). The procedure was also done for full arm losses using a predicted value of <2.

### Mutation analysis

Following library creation as described above, two separate PCRs were designed to selectively enrich the Watson or Crick strand. Both PCRs used the same gene-specific primer, but each used a different anchoring primer. PCR duplicates derived from each strand could be distinguished by the orientation of the insert relative to the exogenous exogenous unique identifier (UID). Sequencing reads underwent initial processing by extracting the first 14 nucleotides as the exogenous barcode sequence (UIDs) and masking adapter sequencing Picard’s IlluminaBasecallsToSam (http://broadinstitute.github.io/picard). Reads were then mapped to the hg19 reference genome using BWA-MEM (*74*) and sorted by barcode sequence using Samtools (*73*). Duplex mutations were defined as mutations present in >80% of both the Watson and Crick families with the same UID. We used the following metrics for the interpretation of mutations: Only samples in which 10 *KRAS* template molecules were amplified were assessed; only mutant positions with more than two mutant template molecules were considered as bona fide. Only genomic positions with at least two or more reported annotations in the Catalogue of Somatic Mutations in Cancer (COSMIC) in genome-wide studies and that were confirmed somatic mutations were considered as bona fide; only positions with at least 5× coverage were considered, and only positions at least 30 bp away from the end of the molecule were considered.

### Fluorescence in situ hybridization

A commercially available probe set purchased from Abbott Molecular, composed of LSI *1p36/TP73* (SpectrumOrange) and LSI *1q25/ABL2* (SpectrumGreen), was applied to individual slides, hybridized, and washed according to the Tris/EDTA FISH protocol. This process was repeated using a commercially available probe set purchased by Abbott Molecular composed of LSI 19q13/EHD2 (SpectrumOrange) and 19p13/D19S221 (SpectrumGreen) on adjacent slides. In each case, 50 to100 nuclei from the cancer cells within the section were evaluated in a blinded manner using two independent analysts. When more than 10 signals from a fluorescent probe were observed in a nucleus, the number recorded in table S3 was considered to be 10.

### Statistical analysis

Cohort sample size was not selected for statistical power but based on sample availability. The two-tailed Fisher’s exact test (alpha = 0.05) was used to test the association with chromosome 1q gain and the mutation status of various genes.
