# Supplementary material for: Evidence that extra copies of chromosome 1q play a role in the early phases of pancreatic neoplasia
Source: Sci Adv. 2026 Feb 20;12(8):eadx7501. doi: 10.1126/sciadv.adx7501 (PMC12922755; doi:10.1126/sciadv.adx7501)
Supplement: Supplementary file 1 — Fig. S1 Legends for tables S1 to S9 [file sciadv.adx7501_sm.pdf]

Science Advances
AAAS

Supplementary Materials for

Evidence that extra copies of chromosome 1q play a role in the early phases of pancreatic neoplasia

Christopher Douville *et al.*

Corresponding author: Christopher Douville, cdouvil1@jhmi.edu; Laura D. Wood, ldelong1@jhmi.edu; Bert Vogelstein, vogelbe@jhmi.edu

Sci. Adv. **12**, eadx7501 (2026)
DOI: 10.1126/sciadv.adx7501

The PDF file includes:

Fig. S1
Legends for tables S1 to S9

Other Supplementary Material for this manuscript includes the following:

Tables S1 to S9

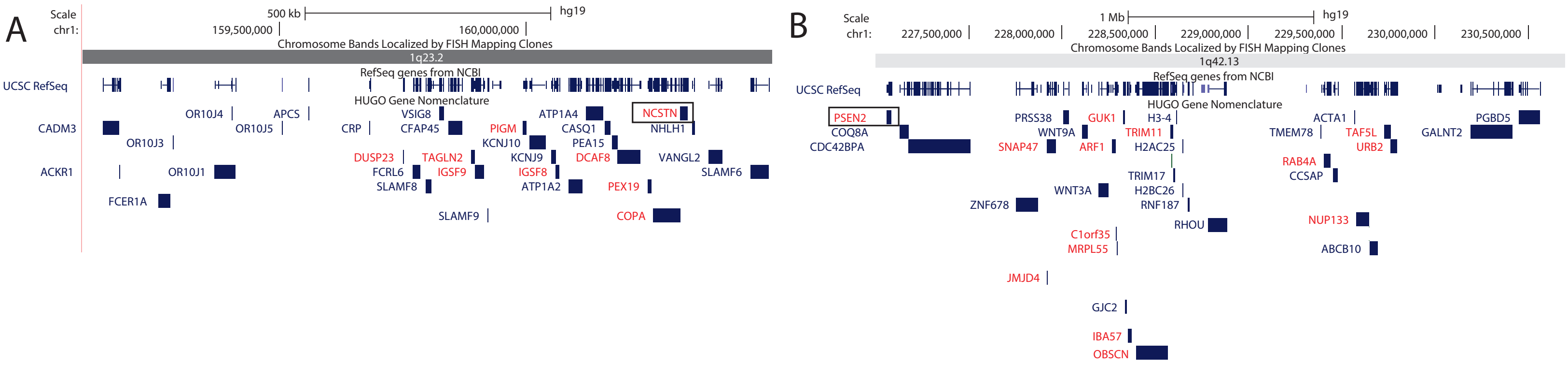

Fig. S1. Visualizations of the genes that produce proteins for the two cytobands of interest. A) 1q23.2 and B) 1q42.13. Genes in red have a statistically significant ($p<1e-5$) correlation between copy number status and gene expression.

Legends for tables S1 to S9

Table S1: Sample and Arm Level Copy Number Information.
Table S2A: Bin Level Copy Number Information (ichor 500kb).
Table S2B: Bin Level Copy Number Information (QDNAseq 500kb).
Table S3: FISH signals per cell.
Table S4: Summary of FISH results and comparison to WGS.
Table S5: Correlation of Copy Number with mRNA Expression for genes in Cytoband 1q23.2.
Table S6: Correlation of Copy Number and mRNA Expression for genes in Cytoband 1q42.13.
Table S7: Genes and regions interrogated by the targeted mutation panel, assuming 75 bp amplicon size.
Table S8: Mutations identified by targeted sequencing in pre-malignant lesions.
Table S9: Summary of mutations and other chromosomal changes and their relationship to chromosome 1q gain (from WGS data).
